# Perceived Safety of Learning Environment and Associated Anxiety Factors during COVID-19 in Ghana: Evidence from Physical Education Practical-Oriented Program

**DOI:** 10.3390/ejihpe12010003

**Published:** 2022-01-01

**Authors:** Frank Quansah, John Elvis Hagan, Francis Sambah, James Boadu Frimpong, Francis Ankomah, Medina Srem-Sai, Munkaila Seibu, Richard Samuel Kwadwo Abieraba, Thomas Schack

**Affiliations:** 1 Department of Educational Foundations, University of Education, Winneba P.O. Box 25, Ghana; fquansah@uew.edu.gh; 2 Department of Health, Physical Education and Recreation, University of Cape Coast, Cape Coast PMB TF0494, Ghana; francis.sambah@stu.ucc.edu.gh (F.S.); james.frimpong@stu.ucc.edu.gh (J.B.F.); 3 Neurocognition and Action-Biomechanics-Research Group, Faculty of Psychology and Sports Science, Bielefeld University, Postfach 10 01 31, 33501 Bielefeld, Germany; thomas.schack@uni-bielefeld.de; 4 College of Public Health, Medical and Veterinary Sciences, James Cook University, Townsville, Queensland, QLD 4811, Australia; 5 Department of Education and Psychology, University of Cape Coast, Cape Coast PMB TF0494, Ghana; francis.ankomah@stu.ucc.edu.gh; 6 Department of Education, SDA College of Education, Asokore-Koforidua P.O. Box AS 18, Ghana; 7 Department of Health, Physical Education, Recreation and Sports, University of Education, Winneba P.O. Box 25, Ghana; mssai@uew.edu.gh (M.S.-S.); mseibu@uew.edu.gh (M.S.); rskabieraba@uew.edu.gh (R.S.K.A.)

**Keywords:** PE students, COVID-19-related anxiety, COVID-19 information, learning environment, safety perception, physical education

## Abstract

The outbreak of COVID-19 led to the swift migration to alternate instructional delivery models and pedagogical practices in educational institutions. This study examined the perceived safety of the learning environment and associated anxiety factors among physical education students amidst COVID-19. Using a cross-sectional design, a sample of 638 students drawn purposively and conveniently from a public university in Ghana completed a self-developed questionnaire. Frequency counts, percentages, and ordered logistic regression were used to analyze the data. Findings of the study showed that students perceived the practical lesson environment as unsafe, with self-reported moderate to high levels of anxiety during their practical lessons. The ordered logistic regression results revealed that varied factors such as age, COVID-19 information platforms, certainty about personal safety, and adequacy of preparation to manage COVID-19 cases were associated with anxiety. The study concluded that an unsafe practical physical education learning environment increases the anxiety levels of students. Academic departments/units should provide periodic interventions (e.g., positive self-talk, mental rehearsal, cognitive restructuring) and counseling services for students amidst the ongoing pandemic to help moderate situational-specific anxiety. In addition, key to the management of students’ anxiety is the provision of a safe and supportive school environment, including the provision of adequate personal protective equipment for practical lessons by school authorities.

## 1. Introduction

Since the global outbreak of SARS-CoV-2 was declared a pandemic [1], numerous measures (e.g., isolating the population, avoiding crowds, and intensification of healthy hygiene behaviors such as proper handwashing, social distancing, confinement, travel restrictions) have been implemented to effectively combat COVID-19 to preserve lives [2]. The key impact of COVID-19 restrictions was the closure of schools, postponement, and/or cancellation of all campus-related academic and social activities, disrupting over 1.2 billion students’ academic pursuits worldwide [3,4]. To continue a pedagogical connection with students, universities had to swiftly migrate their programs and courses to alternate instructional delivery models in all fields through new pedagogical practices [5]. This unprecedented shift posed new challenges, which included the development of alternate studying approaches and self-directed management of the studies for students with widespread uncertainty, anxiety, and panic [3]. For physical education (PE), unlike the teaching of other subjects, the practical didactic design component through visual and manual guidance constitutes nearly between 60% and 70% of the overall study program [6,7,8,9]. Therefore, the adaptation to lectures using a hybrid system to provide authentic planning and modifications to the PE curriculum has become burdensome [10], especially adjusting to new teaching and assessment procedures [8,9]. For both students and teachers, the perception of getting infected with the coronavirus from the learning environment threatens not only their psychological well-being but also their physical, intellectual, emotional, and occupational well-being [11]. Although the current imposition of fewer restrictions due to a decline in case counts across many societies as a result of numerous interventions, the perception of safety measures amidst teaching and learning among students, especially in subjects such as PE, is still a major concern [12]. According to Varea and co-workers [12], the future of PE is still uncertain given the detrimental consequences linked with group activities and ensuring greater personal measures (e.g., space) for each student.

Extant literature has already reported the impact of such pandemics on students’ psychology [13], with university students being at a higher risk during such outbreaks for depression and anxiety [14,15]. COVID-19-related studies have also shown that students’ levels of stress, anxiety, and depression worsened compared to that before the pandemic [16,17]. For example, Cao et al. [16] reported that worries over economic impact and deferments in academic activities were positively related to anxiety symptoms. Students’ reported concerns about their academic performance and declined social interaction because of the pandemic have also been established [18]. Despite numerous studies investigating the impact of COVID-19 on university students’ mental health, economics, social, education, and related stress, anxiety, and depression reactions in several countries [16,19,20,21,22,23,24], the perception of safety measures and the impact of anxiety on students may vary from one country to another, including subject-specific needs [8]. Country-specific data suggest that, as of 5 November 2021, Ghana had a total of 130,608 cases with 128,131 recoveries, 37 severe, 12 critical, 1203 deaths, and 1274 active cases per 100,000 population. This trend (<1.0%) in Ghana is considerably lower, compared to 3.3% of all case fatality and 4.3% of mortality rates reported in the African sub-region and globally [25]. Though about 3,188,144 doses of AstraZeneca, Sputnik-V, Moderna, and Johnson & Johnson vaccines have been administered to some Ghanaians [25], the perceived safety learning environment and potential anxiety reactions of students cannot be underestimated [19].

In Ghana, like other developing countries, the teaching-learning environment amidst COVID-19 is further compounded by a lack of adequate personal protective equipment (PPEs), teaching and learning materials and infrastructure, facilities, technical equipment, and other logistical support [4,10,26,27,28]. Hence, the psychological pressure and burden could be enormous for students and lecturers in light of the ongoing pandemic and context-specific inadequacies [29,30]. Psychological situations (e.g., perceived uncertainty) play a significant role in the implementation of effective public health interventions in pandemic control and prevention such as risk and safety assessment, whereas anxiety also serves as a key factor in the success or failure of these proposed measures [31]. Moreover, other variables such as age, sex, and religion play a critical role in understanding the prevalence of anxiety among any population. Srem-Sai et al. [32] reported a significant association between religion and emotions of student-athletes, with Muslims reporting higher levels of anger than Christians. Other scholars have discovered significant differences in the exhibition of positive and negative emotions on the basis of religion [33,34,35,36,37,38]. In a multi-national study by Prieto and Diener [35], religion (i.e., Christian, Hindu, Muslim, Buddhism, and Jew) was reportedly linked with emotional beliefs and experiences. With regards to sex and age, several other researchers have also shown that sex and age are significant predictors of stressful experiences [39,40,41,42].

Therefore, examining perceived safety in the PE learning environment and associated anxiety factors during the ongoing pandemic could provide scientific guidance for consideration in the re-design of the PE curriculum formulation in Ghana amidst the ongoing pandemic. Moreover, assessing PE students’ psychological reactions to COVID-19 and identifying associated factors could help the implementation of appropriate measures aimed at promoting students’ well-being during this unprecedented period. Specifically, the present study examined: 1. the extent to which PE students perceived the safety of the learning environment during practical lessons amidst COVID-19; 2. anxiety levels of PE students during practical lessons amidst COVID-19; and 3. factors associated with anxiety levels of PE students during practical lessons amidst COVID-19.

## 2. Materials and Methods

### 2.1. Research Approach

The quantitative research approach via a cross-sectional design was employed for this research. This approach was chosen to obtain knowledge and understanding through statistical lenses [43]. Per the objectives of the study, particularly examining the association between COVID-19 anxiety and other variables such as age, sex, and perceived safety, the quantitative approach was the most appropriate [44]. Most importantly, this approach was selected because the research problem required the results to be generalized from the sample of students selected to the population [45]. This approach was because the COVID-19 affected virtually every university student [13,14,15], and thus, sampling more students for such a study was prudent. Several similar studies have supported the use of a quantitative approach for studies of this nature [19,21,22].

### 2.2. Participants’ Selection

The study covered students in a public university in Ghana that run a PE program. The PE students in the University of Education, Winneba (UEW) were purposively chosen as the target group. This selection was because UEW had the overwhelming majority of the regular PE student population. Thus, regular PE students in the Department of Health, Physical Education, Recreation and Sports (HPERS) of UEW-Ghana became the focus of the study. The study conveniently recruited 638 participants from the targeted department to participate in this study. The Institutional Review Board (IRB) of the University of Cape Coast (UCC) approved the survey procedure after all ethical standards were duly met and participants signed consent forms to declare their willingness to be involved in the study.

### 2.3. Instrumentation

A questionnaire with three sections was used for the data collection. The first part of the instrument had demographic variables, namely: age, sex, religion, years spent in university, prior tertiary education, and COVID-19 information platforms. The second part of the instrument had 5-item measures related to the participants’ perceived feeling of safety during PE practical lessons amidst COVID-19. This 5-item perceived feeling of safety proxies were based on the following: (a) feeling of certainty, (b) availability of personal protective equipment (PPEs), (c) comfortability about their participation in class, (d) adequacy of COVID-19 education, and (e) adequacy of school’s preparation toward managing suspected cases. The specific items were: “*I feel certain about the safety of the environment during practical lessons*”, “*There is the availability of necessary personal protective equipment when having practical lessons*”, “*I am comfortable participating in practical lessons during the COVID-19 pandemic*”, “*There is adequate education in my institution on how to protect myself from contracting the virus*”, and “*There is adequate preparation by my school toward managing suspected cases of COVID-19*”. Responses were dichotomous, ‘*Yes*’ or ‘*No*’. The KR 21 reliability estimate of the items was 0.70.

The last section of the questionnaire had 6-items soliciting information on anxiety. The items had a preamble, which read: “indicate the extent to which you have experienced the following symptoms during practical lessons amidst COVID-19 in the past month, including today”. The anxiety items were, “*I feel unsteady*”, “*I fear the worst happening*”, “*I feel very much concerned*”, “*I have self-doubts*”, “*I feel unrelaxed*”, and “*I feel nervous*”. The response options for the item comprised 0–3 (0—*not at all*, 1—*somewhat*, 2—*moderately*, 3—*very much so*). The 6 items on anxiety were developed by the researchers out of Beck et al.’s [46] anxiety scale by using the items that reflected non-clinical symptoms. Those 6-items were validated using confirmatory factor analysis. The reliability estimate using the omega ω estimation procedure was 0.71.

### 2.4. Data Collection Procedure

Following the ethical clearance, with a reference number: UCCIRB/EXT/2020/25 by IRB of UCC, approval was granted by the head of department (HOD), HPERS-UEW, to allow students to participate in the study after official permission was sought from him. The researchers organized a meeting including all students in the department to specifically provide them with a comprehensive briefing on the aim of the survey and other related matters. Participants who were willing and ready were assembled at one of the lecture halls, and the survey instrument was discussed with them to respond to the items appropriately while establishing a suitable rapport with all participants. Aside from assuring participants of keeping their responses confidential and anonymous, participants were also made to understand that only the researchers would have access to the responses they would provide.

Participants were further informed that they could voluntarily decide to continue to respond to or opt-out of the study at any given time without any penalty. All COVID-19 safety protocols, including the wearing of nose masks, providing and ensuring handwashing with soap, hand sanitizing, and social distancing, were strictly observed. The questionnaires were distributed directly to the study participants at least 30 min before a lecture or just after the lecture to either respond to them immediately or later if they so wished. Many of the participants responded to the survey items immediately and were collected by the researchers. The researchers collected all the survey instruments that were responded to by the participants, sealed them in brown envelopes, and kept them with one of the researchers. The data collection process lasted for two weeks.

### 2.5. Data Analysis

The data were first screened and cleaned for data entry errors and by running a descriptive analysis of all the items. Frequency counts and percentages were used to analyze the demographic characteristics of respondents and data on research objective 1 (which aimed at exploring the PE students perceived the safety of the learning environment during practical lessons amidst COVID-19). Using the mean scores of participants across the items, the frequency distribution of the various levels of anxiety was presented. Ordinal logistics regression analysis was performed to assess the associated anxiety factors among PE students during practical lessons amidst COVID-19. Before choosing the ordinal logistics regression, the multiple regression analysis was preferred; however, the normality assumption was violated. Consequently, logistic regression analysis (non-parametric tool) was used based on the recommendations in statistics literature [47]. This was also supported by other empirical studies, which treated the outcome variable as ordinal, although the data on that variable was taken in a continuous form [20,48]. This statistical approach was chosen because of the ordinal nature of the criterion variable [44]. As an assumption, the test of parallel lines analysis was conducted for the overall model, and the results showed that a violation of the assumption, *p* < 0.001. Further, the inspection was performed by examining the global significance (from the test of parallel lines) associated with the predictors, which revealed that seven out of ten predictors did not violate the assumption. Therefore, the ordinal logistics regression was selected over multinomial regression to address the last objective [49].

## 3. Results

### 3.1. Demographic Characteristics of PE Students

The demographic characteristics of the PE students were explored (see Table 1). The personal details surveyed include age, sex, religion, years spent in university, prior tertiary education, and COVID-19 information platforms.

The data, as displayed in Table 1, showed that most of the participants for this study were between the age range of 20–24 years (42%). Whereas few of them were between 25 and 29 years, quite a number of them were within 30–34 years. There were more male participants (72.1%) than females (27.9%). About two-thirds of the sample were Christians (65.8%), and a few of them were atheists (0.9%). The data further showed that about 45.5% of the participants had spent approximately two years at the university, 26.3% had spent three years, 25.1% had spent four years, and only 20 of them had spent a year or less (3.1%). Again, more than half of the sample had received tertiary education before they entered the university (certificate (23.2%) and diploma (49.2%)). A total of 178 of them, however, did not report any history of attaining a tertiary degree (27.6%).

Study participants reported on the platforms or sources where they obtained the COVID-19 information. It is instructive to note that social media (e.g., Facebook, Twitter, WhatsApp) were the notable sources of information about COVID-19 were obtained (29%). This was followed by TV (23.8%) and radio (21.1%). Professional platforms and newspapers/magazines were the least sources of COVID-19 information reported by the participants.

### 3.2. PE Students Perceived Safety of Learning Environment during Practical Lessons Amidst COVID-19

This research explored the extent to which participants felt safe during practical lessons during the COVID-19. The details of the responses are shown in Table 2.

Responses from the participants, as presented in Table 2, showed that the majority of the students were not certain about their safety during practical lessons (54.2%). Although the students acknowledged that there was sufficient COVID-19 education on how to protect themselves from contracting the virus (77.1%), most of them reported non-availability of or inadequacy of PPEs during practical lessons (51.1%). Further results revealed that students perceived the school’s preparation toward suspected COVID-19 cases as inadequate (68%). The students also reported that they were not comfortable participating in practical lessons during the COVID-19 (55.5%).

Generally, the students perceived the practical lesson environment as unsafe. This conclusion is drawn due to the students’ reported level of uncertainty about their safety, inadequate PPEs, and discomfort during PE practical lessons, as well as the perceived inadequate preparations by their schools toward managing COVID-19 cases.

### 3.3. Anxiety Levels of PE Students during Practical Lessons Amidst COVID-19

The reported anxiety levels of PE students during practical lessons were explored. The mean of means for each participant was computed, and scores between 0 and 3 were obtained accordingly. Values between 0 and 1.4 were categorized as low anxiety, scores greater than 1.4 and less than 2.5 were classified as moderate anxiety, and high anxiety participants were defined as having scores greater than or equal to 2.5. These cut-offs were proposed by Beck et al. [46]. The frequency distribution of these participants based on the classification is shown in Table 3.

The results, as presented in Table 3, show that a greater proportion of the PE students reported a moderate level of anxiety during practical lessons (51.7%). About one-fourth of the participants reported high anxiety levels during practical lessons (25.1%). A relatively few number of participants reported low levels of anxiety (23.2%). Largely, the PE students reported moderate to high levels of anxiety during practical lessons (76.8%).

### 3.4. Factors Associated with Anxiety Levels of PE Students during Practical Lessons Amidst COVID-19

The researchers further examined the associated anxiety factors during practical lessons amidst COVID-19. The criterion variable was anxiety levels: low, moderate, and high levels. Ten predictors were used for the model: age, sex, religion, COVID-19 information platforms, years in university, certainty about personal safety, availability of PPEs, comfortable about safety, adequacy of COVID-19 education, adequacy of preparation to manage COVID-19 cases. The details of the ordinal logistic regression are shown in Table 4, Table 5 and Table 6.

The results from the likelihood ratio test showed that there is a significant improvement in the fit of the final model (i.e., the model containing the complete set of predictors) compared to the intercept only model (i.e., the null model), *χ*^2^(19) = 114.304, *p <* 0.001 (see Table 4). It was also found that the set of predictors explained about 16.4% of variations in anxiety levels of students. Both Pearson, *χ*^2^(347) = 1157.327, *p <* 0.001, and deviance goodness-of-fit indices, *χ*^2^(347) = 1138.359, *p <* 0.001, had a contrary result for the final model, indicating poor of fit.

The poor fit from the final model can, however, be explained by the relatively large sample size. To be certain of this, the fit indices associated with the predictors were inspected further (see Table 5). The results showed that out of the 10 indices associated with the predictors, only 3 violated the goodness-of-fit assumption. Since the majority of the individual predictors had a suitable fit, the ordinal regression analysis was maintained [49].

The results from the test of model effect showed that four out of the 10 indicators were significant predictors of anxiety levels of students during practical lessons amidst COVID-19. These variables were age, *χ*^2^(2) = 13.310, *p =* 0.001; COVID-19 information platforms used, *χ*^2^(4) = 13.933, *p <* 0.001; certainty about safety during practical lessons, *χ*^2^(1) = 26.558, *p <* 0.001; and perceived adequacy of school’s preparation toward managing COVID-19 cases, *χ*^2^(1) = 25.723, *p <* 0.001. The details of these predictions are presented in Table 6.

Taking age as a significant predictor, for instance, students who were aged between 25 and 29 years, compared to those between 30 and 34 years, had higher chances of falling into the high anxiety category, *B =* 0.227, *p =* 0.004, *OR =* 1.255, 95% *CI* (1.077, 1.463). With regard to the sources of COVID-19 platforms, students who obtained information from professional platforms, relative to those who obtain information from social media, had lower odds of falling into the high anxiety level class, *B =* −0.243, *p <* 0.001, *OR =* 0.785, 95% *CI* (0.691, 0.891). Similarly, students who fetched COVID-19 information from the TV, compared to those who obtain information from social media, had lower odds of falling into the high-level anxiety category, *B =* −0.191, *p =* 0.005, *OR =* 0.826, 95% *CI* (0.724, 0.943). However, students who obtained information from the radio station had higher odds of falling into the high-level anxiety category, *B =* 0.166, *p =* 0.011, *OR =* 1.181, 95% *CI* (1.039, 1.343).

The results further suggested that students who felt uncertain about their safety during practical lessons amidst COVID-19, compared to those who were certain, had higher odds of falling into the high anxiety level class, *B =* 0.329, *p <* 0.001, *OR =* 1.390, 95% *CI* (1.228, 1.573). It was discovered that the students who were perceived that the institution is prepared for COVID-19 suspected cases, relative to those who perceived that the institution is not prepared, had lower chances of falling into the high anxiety category, *B =* −0.299, *p <* 0.001, *OR =* 0.742, 95% *CI* (0.662, 0.832).

## 4. Discussion

This research sheds light on the perceived safety of the learning environment and associated anxiety factors among PE students taking practical lessons during COVID-19 in Ghana. The study revealed that PE students perceived the practical lesson environment as unsafe. This assertion is drawn due to the students reported level of uncertainty about their safety, inadequate PPEs, and discomfort during PE practical lessons, as well as the perceived inadequate preparations by their schools toward managing COVID-19 cases. Though there are no probable similar findings to contrast the present finding, however, where uncertainty about evidence or knowledge to inform practice, rights of these students in terms of safety and preferences, as well as other expectations, can influence the environmental climate and the settings in which they find themselves [50]. The novel nature of this virus introduced some hysteria among people as most personal protective and preventive messages could not be verifiably ascertained, coupled with the lack of and poor implementation and enforcement of the COVID-19 protocols. This finding implies that once learners perceive the learning environment as unsafe, it may lead to learner attrition, lack of confidence to participate due to fear of contracting the virus, and increased levels of anxiety, which may increase their susceptibility to sustaining latent injuries [51,52,53]. It was adduced from the present findings that the conduciveness of safe school climate during this COVID-19 era may positively impact the management of COVID-19 related anxieties [54,55]. This assertion has been affirmed in this study where PE students who were certain about their safety during practical lessons amidst COVID-19 were more likely to experience lower levels of anxiety, as compared to those who were uncertain about their safety [56].

Moreover, another far-reaching evidence is the inadequate preparedness of their institutions to manage cases of COVID-19. The consequence of this finding could trigger anxiety-related behavior due to fear and uncertainty of the unknown should one contract the virus. This was evident in both developed and developing nations as governments and institutions became overwhelmed by the level of investment needed to adequately manage and contain the disease [25,57]. The reason most governments, as part of the prevention process, closed down schools, introduced virtual classrooms for learning, designated case management centers, among others [6,30,57,58]. However, when institutions are well prepared with adequate infrastructure to manage COVID-19 cases, it becomes a buffer against anxiety. This premise is buttressed in the findings of this study, where the students who perceived that the institution is prepared for managing suspected COVID-19 cases were more likely to experience lower levels of anxiety as compared to those who perceived otherwise. In this light, in order to build students’ resilience and enhance their adaptability skills during this period of COVID-19, governments and educational institutions must put up case management centers on their school campuses [57].

Largely, findings of this research show that PE students reported moderate to high levels of anxiety during practical lessons, with more than 50% of the sample falling within the moderate anxiety level. This is not surprising as respondents had earlier indicated that their learning environment was unsafe, added to the inadequate preparedness of their schools to manage COVID-19 cases. This is well supported by the previous finding that practicum sessions create a high-risk environment and demonstrate as a risk factor for stress and anxiety [59]. This might have been one of the reasons for the high anxiety reported in this present research since PE involves practical instructional settings for students’ academic interactions. Further, the early strain of the COVID-19 pandemic started as a virulent agent causing high morbidity and mortality with media reportage inconsistent with the mode of spread and the best preventive protocols [60]. This, therefore, created fear and situation-specific anxiety across all demographic groups [61,62]. The moderate to high anxiety noted in the finding could be inferred that the student may be at different levels of knowledge and awareness of the appropriate comprehensive scientific information about the etiology and spread of the virus [1]. Academic departments/units should create periodic opportunities for guidance and counseling services during pandemic situations to help mediate situational-specific anxiety. In addition, educators, parents, and educational institutions should endeavor to provide a safe and supportive school environment [10,63].

Furthermore, this research revealed that younger students, as compared to older ones, were more likely to experience higher levels of anxiety during practical lessons amidst COVID-19. This finding is reflected in Islam et al.’s [48] study, which found younger students expressing higher anxiety compared to older students. One probable reason is that younger students may have higher risk perception, possibly due to health risk behavior indulgence and low self-efficacy toward adhering to the safety protocols. It is also possible that the older students may have better coping mechanisms in managing their anxieties, although this claim needs further investigation. Interventions to allay anxiety among students should target younger students.

COVID-19 information has dominated both traditional and electronic media platforms in recent times, with some serving suitable purposes and others serving hoax messages to the general public [1,30,64,65]. The channel from which one receives COVID-19 related information can influence and raise one’s anxiety level, as reported in other previous studies [30,66]. This aligns with our finding where students who obtained COVID-19 information from social media and radio were more likely to experience high levels of anxiety as compared to those who fetch COVID-19 information from professional platforms and TV. This finding is reliable because the information from radio and social media platforms may not be censored and peer-reviewed compared to information from professional platforms [66]. This finding is similar to a previous study where Ko et al. [67] observed that participants who received COVID-19 information from non-professional sources reported poorer psychological well-being. We suggest that school authorities should establish reliable news portals to feed students with reliable and credible information concerning COVID-19.

### 4.1. Strengths and Limitations

Because study participants were purposively and conveniently recruited from only one public university and a practical-oriented program in Ghana, the representativeness of the sample is restricted. Therefore, the generalization of the findings to a larger student population and other study programs in Ghana should be noted with caution. Moreover, due to the cross-sectional design used, the causal association between selected factors and anxiety levels could not be established. Like most surveys, study participants are likely to either under or over report on identified outcomes due to inherent social desirability concerns often associated with self-reported measures. Despite these limitations, present findings provide empirical evidence on students’ psychological health in Ghana, adding to the sparse literature during this pandemic as well as offering useful information for appropriate interventions. Future research should conduct a detailed longitudinal analysis of what factors explain this high perceived risk among the larger students’ population in Ghana. Such studies could incorporate institutional administrators and teachers as participants to understand the preparations to validate the claims of the students in this present study.

### 4.2. Practical Implications

Present findings have practical implications for students’ mental health and didactic design through appropriate interventions that seek to promote a safe and supportive learning environment, including functional coping strategies to manage reported anxiety experiences during the ongoing pandemic. The reported moderate-high COVID-19 anxiety and associated factors highlight the roles required of educational personnel such as PE teachers, school counselors, and psychologists toward students’ psychological adjustment and pedagogical practices conducive to learning. The identification of COVID-19 management resources (e.g., PPEs) and the use of creative or innovative learning approaches (e.g., hybrid modules) by PE teachers could help students attain their learning outcomes while maintaining safety practices and standard teaching as well as learning conditions. It is also imperative for educational institutions of higher learning in Ghana to provide reliable news outlets or portals for credible COVID-19 information as a control strategy for the management of students’ anxiety levels, especially among younger age groups.

## 5. Conclusions

This study has revealed that PE students perceived the practical lesson environment as unsafe and reported moderate to high levels of anxiety during practical lessons. These findings imply that the practical PE lesson environment is not safe and serves as a potential portal for COVID-19 acquisition and transmission while also increasing the anxiety levels of PE students. The study also revealed that PE students who were certain about their safety during practical lessons amidst COVID-19 and those who perceived that the institution was prepared for managing suspected COVID-19 cases were more likely to experience lower levels of anxiety compared with their peers. Younger students and those who obtained COVID-19 information from social media and radio were more likely to experience high levels of anxiety. Academic departments/units should provide periodic resilient and adaptable interventions (e.g., positive self-talk, mental rehearsal, cognitive restructuring) and counseling services for students amidst the ongoing pandemic to help moderate situational-specific anxiety, especially among younger students. In addition, key to the management of students’ anxiety is the provision of a safe and supportive school environment, including the provision of adequate PPEs for practical lessons by school authorities through governmental assistance and other private stakeholders. School authorities should establish reliable news portals to feed students with reliable and credible information on COVID-19.

## Figures and Tables

**Table 1:** Personal characteristics of the respondents.

Variables	Categories	Frequency Counts	Percent
Age range	20–24 years	268	42.0
	25–29 years	124	19.4
	30–34 years	246	38.6
	>34 years	--	--
Sex	Male	460	72.1
	Female	178	27.9
Religion	Christian	420	65.8
	Muslim	180	28.2
	Traditionalist	32	5.0
	Atheist	6	0.9
Years in university	1 year or less	20	3.1
	2 years	290	45.5
	3 years	168	26.3
	4 years	160	25.1
Prior education (tertiary)	Certificate	148	23.2
	Diploma	314	49.2
	None	178	27.6
Usual COVID-19 info platforms	Professional platforms	87	13.7
	Social media	185	29.0
	Newspapers/magazine	79	12.4
	Radio	135	21.1
	Television (TV)	152	23.8

**Table 2:** Responses on perceived safety of learning environment.

Items	Yes, *n* (%)	No, *n* (%)
Feeling of certainty about the safety of the environment during practical lessons	292 (45.8)	346 (54.2)
Availability of necessary personal protective equipment when having practical lessons	312 (48.9)	326 (51.1)
Comfortability about participating in practical lessons during COVID-19	284 (44.5)	354 (55.5)
Adequacy of education on how to protect themselves from contracting the virus	492 (77.1)	146 (22.9)
Adequacy of the school’s preparation toward managing suspected cases of COVID-19	204 (32.0)	434 (68.0)

**Table 3:** Proportions of PE students with low, moderate and high anxiety levels during PE practical lessons.

Anxiety Levels	Score Range	Frequency	Percent
Low anxiety	0–1.4	148	23.2
Moderate anxiety	>1.4–<2.5	330	51.7
High anxiety	≥2.5	160	25.1
Total	--	638	100.0

**Table 4:** Model fit indices of the ordinal logistics regression analysis.

Model	−2 Log Likelihood	Chi-Square	df	Sig.
Model Fitting Information				
Intercept Only	1268.550			
Final	1154.246	114.304	19	<0.001
Goodness of Fit				
Pearson	--	1157.327	347	<0.001
Deviance	--	1138.359	347	<0.001

Cox and Snell = 0.164; Nagelkerke = 0.188.

**Table 5:** Global significance indices associated with the predictors.

	Likelihood Ratio Chi-Square	df	Sig.	Fit Indices	
				Chi-Square	Sig.
(Intercept)	87.471	1	<0.001		
Age	13.310	2	0.001	0.282	0.868
Sex	2.682	1	0.101	0.001	0.997
Religion	1.363	3	0.714	9.791	0.020
Use of COVID-19 information platforms	13.933	4	<0.001	0.186	0.666
Years spent in university	2.472	3	0.480	17.195	0.001
Certainty about the personal safety	26.558	1	<0.001	7.067	0.008
Availability of PPEs	0.521	1	0.471	1.329	0.249
Comfortable about safety	0.178	1	0.673	0.087	0.768
Adequacy of COVID-19 education	0.001	1	0.969	2.932	0.087
Adequacy of school’s preparation	25.723	1	<0.001	2.143	0.143

Dependent variable: anxiety levels. Model: (intercept), age, sex, religion, COVID-19 information platforms, years in university, certainty about personal safety, availability of PPEs, comfortable about safety, adequacy of COVID-19 education, adequacy of preparation to manage COVID-19 cases.

**Table 6:** Parameter estimates of the predictors of students’ anxiety levels during COVID-19.

Parameter	B	Std. Error	95% Wald CI		Hypothesis Test			Exp(B)	95% Wald CI Exp(B)	
			Lower	Upper	Wald Chi-Square	df	Sig.		Lower	Upper
(Intercept)	0.684	0.2949	0.106	1.262	5.376	1	0.020	1.981	1.112	3.532
Age										
20–24 years	−0.039	0.0710	−0.178	0.100	0.303	1	0.582	0.962	0.837	1.105
25–29 years	0.227	0.0781	0.074	0.380	8.464	1	0.004 *	1.255	1.077	1.463
30–34 years (ref)	0 ^a^							1		
Sex										
Male	0.101	0.0619	−0.020	0.223	2.688	1	0.101	1.107	0.980	1.249
Female (ref)	0 ^a^							1		
Religion										
Christian	0.284	0.2770	−0.259	0.827	1.052	1	0.305	1.329	0.772	2.287
Muslim	0.310	0.2781	−0.235	0.855	1.241	1	0.265	1.363	0.790	2.351
Traditionalist	0.263	0.2933	−0.312	0.838	0.804	1	0.370	1.301	0.732	2.311
Atheist (ref)	0 ^a^							1		
COVID-19 information platforms										
Professional platforms	−0.243	0.0646	−0.369	−0.116	14.086	1	0.001 *	0.785	0.691	0.891
Television (TV)	−0.191	0.0677	−0.324	−0.058	7.969	1	0.005 *	0.826	0.724	0.943
Newspaper/magazines	−0.025	0.0754	−0.173	0.123	0.109	1	0.742	0.975	0.842	1.131
Radio	0.166	0.0656	0.038	0.295	6.436	1	0.011 *	1.181	1.039	1.343
Social media (ref)	0 ^a^							1		
Years at the university										
1 year or less	−0.057	0.1553	−0.362	0.247	0.137	1	0.711	0.944	0.696	1.280
2 years	0.106	0.0793	−0.049	0.261	1.786	1	0.181	1.112	0.952	1.299
3 years	0.061	0.0759	−0.088	0.210	0.650	1	0.420	1.063	0.916	1.234
4 years (ref)	0 ^a^							1		
Feeling of certainty about safety										
Uncertain	0.329	0.0632	0.205	0.453	27.119	1	<0.001 *	1.390	1.228	1.573
Certain (ref)	0 ^a^							1		
Availability of PPEs										
Not available	−0.041	0.0567	−0.152	0.070	0.521	1	0.470	0.960	0.859	1.073
Available (ref)	0 ^a^							1		
Comfortability in school										
Not comfortable	0.026	0.0614	−0.094	0.146	0.178	1	0.673	1.026	0.910	1.157
Comfortable (ref)	0 ^a^							1		
Adequacy of COVID-19 education										
Not educated	−0.003	0.0679	−0.136	0.130	0.001	1	0.969	0.997	0.873	1.139
Educated (ref)	0 ^a^							1		
Workplace prepared for cases										
Workplace prepared	−0.299	0.0583	−0.413	−0.184	26.249	1	<0.001 *	0.742	0.662	0.832
Workplace not prepared (ref)	0 ^a^							1		
(Scale)	0.400 ^b^	0.0224	0.358	0.446						

Dependent variable: anxiety level; Model: (intercept), age, sex, religion, COVID-19 information platforms, years in university, certainty about personal safety, availability of PPEs, comfortable about safety, adequacy of COVID-19 education, adequacy of preparation to manage COVID-19 cases; * significant at *p* < 0.05 level. ^a^ set to zero parameter; ^b^ fixed at the displayed value.

## Data Availability

The data are available upon reasonable request through the corresponding author.

## References

[1] World Health Organization (2020). Coronavirus Disease (COVID-19) Situation Report–102. https://www.who.int/docs/defaultsource/coronaviruse/situationreports/.

[2] Cruz M.P., Santos E., Cervantes M.V., Juárez M.L. (2020). COVID-19, una emergencia de salud pública mundial. Rev. Clin. Esp..

[3] Capone V., Caso D., Donizzetti A.R., Procentese F. (2020). University student mental well-being during COVID-19 outbreak: What are the relationships between information seeking, perceived risk and personal resources related to the academic context?. Sustainability.

[4] UNESCO (2020). Adverse Consequences of School Closures.

[5] Crawford J., Butler-Henderson K., Jurgen R., Malkawi B.H., Glowatz M., Burton R., Magni P., Lam S. (2020). COVID-19: 20 countries’ higher education intra-period digital pedagogy responses. J. Appl. Learn. Teach..

[6] Almonacid-Fierro A., Souza de Carvalho R., Castillo-Retamal F., Almonacid M. (2021). The practicum in times of COVID-19: Knowledge developed by future physical education teachers in virtual modality. Int. J. Learn. Teach. Educ. Res..

[7] Lander N., Eather N., Morgan P.J., Salmon J., Barnett L.M. (2017). Characteristics of teacher training in school-based physical education interventions to improve fundamental movement skills and/or physical activity: A systematic review. Sports Med..

[8] Mocanu G.D., Murariu G., Georgescu L., Sandu I. (2021). Investigating the Attitudes of First-Year Students of the Faculty of Physical Education and Sports of Galati towards Online Teaching Activities during the COVID-19 *Pandemic*. Appl. Sci..

[9] Mocanu G.D., Murariu G., Iordan D.A., Sandu I., Munteanu M.O.A. (2021). The Perception of the Online Teaching Process during the COVID-19 Pandemic for the Students of the Physical Education and Sports Domain. Appl. Sci..

[10] UNICEF (2020). UNICEF and Microsoft Launch Global Learning Platform to Help Address COVID-19 Education Crisis. https://www.unicef.org/pressreleases/unicef-and-microsoft-launch-global-learning-platform-help-address-covid-19-education.

[11] Patrick S.W., Henkhaus L.E., Zickafoose J.S., Lovell K., Halvorson A., Loch S., Letterie M., Davis M.M. (2020). Well-being of parents and children during the COVID-19 pandemic: A national survey. Pediatrics.

[12] Varea V., González-Calvo G. (2020). Touchless Classes and Absent Bodies: Teaching Physical Education in Times of COVID-19. Sport Educ. Soc..

[13] Aktekin M., Karaman T., Senol Y.Y., Erdem S., Erengin H., Akaydin M. (2001). Anxiety, depression and stressful life events among medical students: A prospective study in Antalya, Turkey. Med. Educ..

[14] Zivin K., Eisenberg D., Gollust S.E., Golberstein E. (2009). Persistence of mental health problems and needs in a college student population. J. Affect. Disord..

[15] American College Health Association (2018). American College Health Association National College Health Assessment II: Reference Group Executive Summary Fall 2017.

[16] Cao W., Fang Z., Hou G., Han M., Xu X., Dong J., Zheng J. (2020). The psychological impact of the COVID-19 epidemic on college students in China. Psychiatry Res..

[17] Elmer T., Mepham K., Stadtfeld C., Capraro V. (2020). Students under lockdown: Comparisons of students’ social networks and mental health before and during the COVID19 crisis in Switzerland. PLoS ONE.

[18] Son C., Hegde S., Smith A., Wang X., Sasangohar F. (2020). Effects of COVID-19 on college students’ mental health in the United States: Interview survey study. J. Med. Internet Res..

[19] Arënliu A., Bërxulli D., Perolli-Shehu B., Krasniqi B., Gola A., Hyseni F. (2021). Anxiety and depression among Kosovar university students during the initial phase of outbreak and lockdown of COVID-19 pandemic. Health Psychol. Behav. Med..

[20] Guan Y., Deng H., Zhou X. (2020). Understanding the impact of the COVID-19 pandemic on career development: Insights from cultural psychology. J. Vocat. Behav..

[21] Hoque M.N., Hannan A., Imran S., Alam M.A., Matubber B., Saha S.M. (2021). Anxiety and Its Determinants among Undergraduate Students during E-learning in Bangladesh Amid COVID-19. J. Affect. Disord..

[22] Khoshaim H.B., Al-Sukayt A., Chinna K., Nurunnabi M., Sundarasen S., Kamaludin K., Baloch G.M., Hossain S.F.A. (2020). Anxiety level of University students during COVID-19 in Saudi Arabia. Front. Psychiatry.

[23] Lee J., Jeong H.J., Kim S. (2021). Stress, anxiety, and depression among undergraduate students during the COVID-19 pandemic and their use of mental health services. Innov. High. Educ..

[24] Vulić-Prtorić A., Selak M.B., Sturnela P. (2020). The psychological distress in students during the COVID-19 crisis: An 8-wave longitudinal study. PsyArXiv.

[25] Ghana Health Service (2021). Ghana’s Outbreak Response Management Update. https://www.ghs.gov.gh/covid19/.

[26] Agormedah E.K., Henaku E.A., Ayite D.M.K., Ansah E.A. (2020). Online learning in higher education during COVID-19 pandemic: A case of Ghana. J. Educ. Technol. Syst..

[27] UNESCO (2020). COVID-19: Educational Disruption and Response.

[28] UNESCO (2020). Universities Tackle the Impact of COVID-19 on Disadvantaged Students.

[29] Bai Y., Lin C.-C., Lin C.-Y., Chen J.-Y., Chue C.-M., Chou P. (2004). Survey of stress reactions among health care workers involved with the SARS outbreak. Psychiatr. Serv..

[30] Pragholapati A. (2020). COVID-19 impact on students. EdArXiv.

[31] Lasheras I., Gracia-García P., Lipnicki D.M., Bueno-Notivol J., López-Antón R., de la Cámara C., Lobo A., Santabárbara J. (2020). Prevalence of Anxiety in Medical Students during the COVID-19 Pandemic: A Rapid Systematic Review with Meta-Analysis. Int. J. Environ. Res. Public Health.

[32] Srem-Sai M., Frimpong J.B., Abieraba R.S.K., Sorkpor R.S., Hagan J.E., Schack T. (2021). Religion as a Function of Self-reported Discrete Emotions Among Elite Student-Athletes Before Competition. Int. J. Psychol. Brain Sci..

[33] Cohen A.B. (2002). The importance of spirituality in well-being for Jews and Christians. J. Happiness Stud..

[34] Hagan J.E. (2021). Investigating Pre-Competition Related Discrete Emotions and Unaccustomed Religious Coping among Elite Student-athletes: Implications for Reflexive Practice. Religions.

[35] Kim-Prieto C., Diener E. (2009). Religion as a source of variation in the experience of positive and negative emotions. J. Posit. Psychol..

[36] Tsai J.L., Miao F.F., Seppala E. (2007). Good feelings in Christianity and Buddhism: Religious differences in ideal affect. Pers. Soc. Psychol. Bull..

[37] Vishkin A. (2021). Variation and consistency in the links between religion and emotion regulation. Curr. Opin. Psychol..

[38] Vishkin A., Bigman Y., Tamir M., Kim-Prieto C. (2014). Religion, emotion regulation, and well-being. Positive Psychology of Religion and Spirituality Across Cultures.

[39] Anshel M.H., Sutarso T., Jubenville C. (2009). Racial and gender differences on sources of acute stress and coping style among competitive athletes. J. Soc. Psychol..

[40] Giacobbi P., Foore B., Weinberg R.S. (2004). Broken clubs and expletives: The sources of stress and coping responses of skilled and moderately skilled golfers. J. Appl. Sport Psychol..

[41] Hoar S.D., Crocker P.R., Holt N.L., Tamminen K.A. (2010). Gender differences in adolescent athletes’ coping with interpersonal stressors in sport: More similarities than differences?. J. Appl. Sport Psychol..

[42] Woodman T.I., Hardy L.E. (2003). The relative impact of cognitive anxiety and self-confidence upon sport performance: A meta-analysis. J. Sports Sci..

[43] Creswell J.W. (2012). Educational Research: Planning, Conducting and Evaluating Quantitative and Qualitative Research.

[44] Tabachnick B.G., Fidell L.S. (2019). Using Multivariate Statistics.

[45] Cohen L., Manion L., Morrison K. (2013). Research Methods in Education.

[46] Beck A.T., Epstein N., Steer R.A. (1988). An inventory for measuring clinical anxiety: Psychometric properties. J. Consult. Clin. Psychol..

[47] Tripepi G., Jager K.J., Dekker F.W., Zoccali C. (2008). Linear and logistic regression analysis. Kidney Int..

[48] Islam M.A., Barna S.D., Raihan H., Khan M.N.A., Hossain M.T. (2020). Depression and anxiety among university students during the COVID-19 pandemic in Bangladesh: A web-based cross-sectional survey. PLoS ONE.

[49] Pallant J. (2010). SPSS Survival Manual: A Step by Step Guide to Data Analysis Using SPSS.

[50] Broom A., Broom J. (2017). Fear, duty and the moralities of care: The Ebola 2014 threat. J. Sociol..

[51] Dong L., Bouey J. (2020). Public mental health crisis during COVID-19 pandemic, China. Emerg. Infect. Dis..

[52] Shigemura J., Ursano R.J., Morganstein J.C., Kurosawa M., Benedek D.M. (2020). Public responses to the novel 2019 coronavirus (2019-nCoV) in Japan: Mental health consequences and target populations. Psychiatry Clin. Neurosci..

[53] Wolf L.J., Haddock G., Manstead A.S., Maio G.R. (2020). The importance of (shared) human values for containing the COVID-19 pandemic. Br. J. Soc. Psychol..

[54] Germani A., Buratta L., Delvecchio E., Mazzeschi C. (2020). Emerging adults and COVID-19: The role of individualism-collectivism on perceived risks and psychological maladjustment. Int. J. Environ. Res. Public Health.

[55] Šrol J., Mikušková E.B., Čavojová V. (2021). When we are worried, what are we thinking? Anxiety, lack of control, and conspiracy beliefs amidst the COVID-19 pandemic. Appl. Cogn. Psychol..

[56] Quansah F., Hagan J.E., Ankomah F., Srem-Sai M., Frimpong J.B., Sambah F., Schack T. (2022). Relationship between COVID-19 Related Knowledge and Anxiety: Moderating Roles of School Climate and Coping Strategies among University Students During COVID-19 Pandemic. Front. Psychol..

[57] Sarkodie B., Asiedu-Bekoe F., Laryea D.O., Ampofo W.K., Phillips R.O., Samba A., Nsiah-Asare A., Asamoah-Baah A., Odame E., Ohene S.A. (2021). Overview of preparedness and response to COVID-19 in Ghana. Ghana Med..

[58] Basheti I.A., Mhaidat Q.N., Mhaidat H.N. (2021). Prevalence of anxiety and depression during COVID-19 pandemic among healthcare students in Jordan and its effect on their learning process: A national survey. PLoS ONE.

[59] Cheung T., Wong S.Y., Wong K.Y., Law L.Y., Ng K., Tong M.T., Wong K.Y., Ng M.Y., Yip P.S. (2016). Depression, anxiety and symptoms of stress among baccalaureate nursing students in hong kong: A cross-sectional study. Int. J. Environ. Res. Public Health.

[60] Rajkumar R.P. (2020). COVID-19 and mental health: A review of the existing literature. Asian J. Psychiatr..

[61] Centers for Disease Control and Prevention (CDC) (2020). Healthcare Personnel and First Responders: How to Cope with Stress and Build Resilience During the COVID-19 Pandemic. https://www.cdc.gov/coronavirus/2019-ncov/hcp/mental-health-healthcare.html.

[62] Weinberg R.S., Gould D. (2019). Foundations of Sport and Exercise Psychology.

[63] Laar R.A., Ashraf M.A., Ning J., Ji P., Fang P., Yu T., Khan M.N. (2021). Performance, Health, and Psychological Challenges Faced by Students of Physical Education in Online Learning during COVID-19 Epidemic: A Qualitative Study in China. Healthcare.

[64] Rahayu R.N., Sensusiyati S. (2020). Analysis of Covid-19 hoax news on social media in Indonesia. J. Econ. Soc. Hum..

[65] Zarocostas J. (2020). How to fight an infodemic. Lancet.

[66] Ahinkorah B.O., Ameyaw E.K., Hagan J.E., Seidu A.A., Schack T. (2020). Rising above misinformation or fake news in Africa: Another strategy to control COVID-19 spread. Front. Commun..

[67] Ko N.Y., Lu W.H., Chen Y.L., Li D.J., Wang P.W., Hsu S.T., Chen C.C., Lin Y.H., Chang Y.P., Yen C.F. (2020). COVID-19-related information sources and psychological well-being: An online survey study in Taiwan. Brain Behav. Immun..

